# H2 influenza A virus is not pathogenic in *Tmprss2* knock-out mice

**DOI:** 10.1186/s12985-020-01323-z

**Published:** 2020-04-22

**Authors:** Ruth Lydia Olga Lambertz, Ingo Gerhauser, Inga Nehlmeier, Sabine Gärtner, Michael Winkler, Sarah Rebecca Leist, Heike Kollmus, Stefan Pöhlmann, Klaus Schughart

**Affiliations:** 1 Department of Infection Genetics, Helmholtz Centre for Infection Research, Braunschweig, Germany; 2 Department of Pathology, University of Veterinary Medicine Hannover, Hannover, Germany; 3 Infection Biology Unit, German Primate Center – Leibniz Institute for Primate Research, Göttingen, Germany; 4 Current Address: Department of Epidemiology, University of North Carolina, Chapel Hill, USA; 5 Faculty of Biology and Psychology, University Göttingen, Göttingen, Germany; 6 University of Veterinary Medicine Hannover, Hannover, Germany; 7 Department of Microbiology, Immunology and Biochemistry, University of Tennessee Health Science Center, Memphis, TN USA

**Keywords:** Influenza a virus, H2 subtype, Host protease, TMPRSS2, Mouse mutant

## Abstract

The host cell protease TMPRSS2 cleaves the influenza A virus (IAV) hemagglutinin (HA). Several reports have described resistance of *Tmprss2*^*−/−*^ knock-out (KO) mice to IAV infection but IAV of the H2 subtype have not been examined yet. Here, we demonstrate that TMPRSS2 is able to cleave H2-HA in cell culture and that *Tmprss2*^*−/−*^ mice are resistant to infection with a re-assorted PR8_HA(H2) virus. Infection of KO mice did not cause major body weight loss or death. Furthermore, no significant increase in lung weights and no virus replication were observed in *Tmprss2*^*−/−*^ mice. Finally, only minor tissue damage and infiltration of immune cells were detected and no virus-positive cells were found in histological sections of *Tmprss2*^*−/−*^ mice. In summary, our studies indicate that TMPRSS2 is required for H2 IAV spread and pathogenesis in mice. These findings extend previous results pointing towards a central role of TMPRSS2 in IAV infection and validate host proteases as a potential target for antiviral therapy.

## Main text

The hemagglutinin (HA) of influenza A virus (IAV) facilitates viral entry into target cells. For this, HA binds to cellular receptors and fuses the viral membrane with a target cell membrane. HA is synthesized as an inactive precursor molecule, HA_0_, and needs to be processed by host proteases into the subunits HA_1_ and HA_2_ in order to acquire membrane fusion competence [1–4]. It has been shown previously that the transmembrane protease serine 2 (TMPRSS2) is required for cleavage of HA of subtypes H1, H7 and H10 in infected mice [5–8]. TMPRSS2 cleaves these HAs at a single monobasic cleavage site [2, 3, 9]. Here, we demonstrate that also the HA from H2 IAV subtypes requires processing by TMPRSS2 in vivo for virus replication and pathogenesis.

**Fig. 1:**
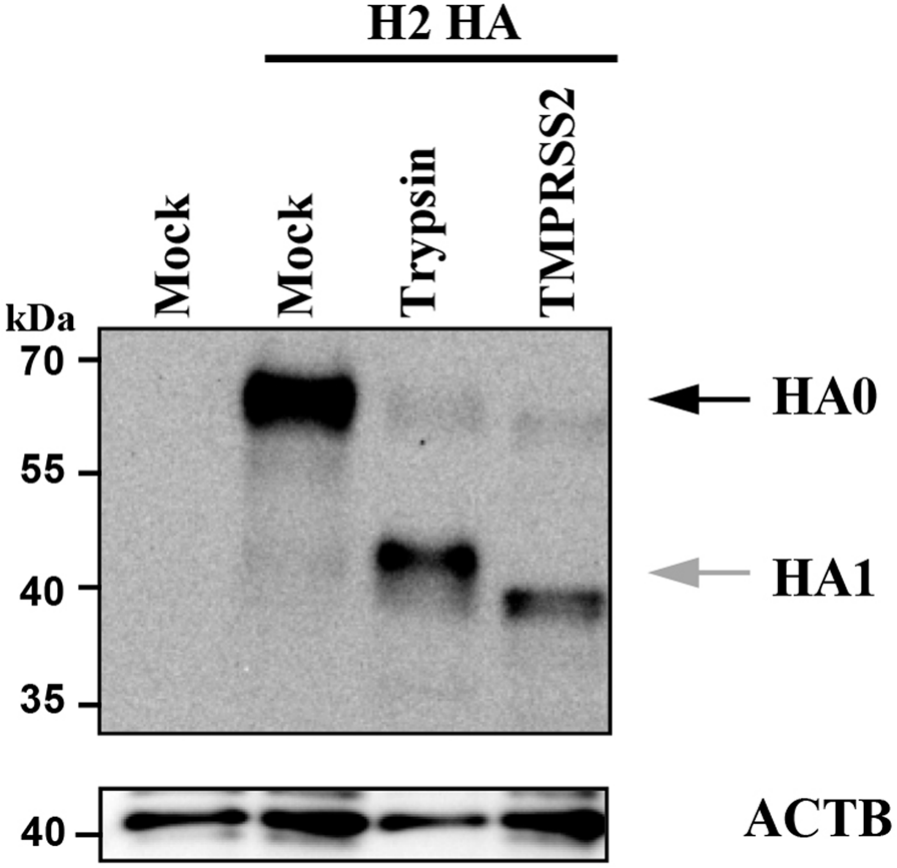
TMPRSS2 cleaves H2-HA. Human embryonic kidney 293 T cells were cotransfected with plasmids encoding H2-HA and plasmids encoding TMPRSS2 of murine origin or empty plasmid (Mock) as negative control. At 48 h post transfection, cells were harvested and treated with either PBS or TPCK trypsin followed by analysis of HA expression by immunoblot, using antiserum raised against H2-HA. Detection of β-actin (ACTB) served as loading control. The results were confirmed in an independent experiment. The black arrow indicates uncleaved HA_0_ (HA0), the grey arrow indicates cleaved HA_1_ (HA1)

First, we investigated the cleavage of HA from subtype 2 IAV (H2-HA) by TMPRSS2 in cell culture. For this, we cotransfected 293 T cells with plasmids encoding H2-HA from A/mallard/Alberta/79/2003 (H2N3) and murine *Tmprss2* [10]. Transfection of empty plasmid served as negative control while treatment of HA expressing cells with trypsin served as positive control. As shown in Fig. 1, trypsin and TMPRSS2 cleaved HA, as determined by the detection of the HA cleavage product HA_1_. The slight differences in the size of the HA_1_ bands produced by TMPRSS2 relative to trypsin have been documented previously and reflect differences in N-glycosylation of HA_1_ [11].

For infection studies in mice, we generated a (7 + 1) re-assorted virus carrying the H2-HA from the A/mallard/Alberta/79/2003 (H2N3) virus on the backbone of A/Puerto Rico/8/34 (H1N1, PR8) virus. In this way, results were independent of other gene segments from the donor bird virus and comparable to other studies in which the HA segment was exchanged and tested on an isogenic PR8 background [8]. For the generation of the PR8_HA(H2) virus, the HA encoding segment 4 from the avian virus was cloned by sequence and ligation independent cloning as described earlier [12] into plasmid pHW-2000 (kindly provided by Robert Webster, St. Jude, Memphis, USA) using primers 5′-gacctccgaagttgggggggAGCAAAAGCAGGGG-3′ and 5′-ttttgggccgccgggttattAGTAGAAACAAGGGTGTTTT-3′. Re-assorted virus was then rescued from plasmids as described earlier [13] with 300 ng of each plasmid, 7.5 μl TransIT-2020 (Mirus) in 250 μl OptiMEM (Gibco) using the H2 encoding plasmid and plasmids containing all other seven segments of PR8 (kindly provided by Robert Webster, St. Jude, Memphis, USA). The resulting virus PR8_HA(H2) virus was propagated in the chorio-allantoic cavity of 10-day-old specific pathogen free (SPF) embryonated chicken eggs (Charles River Laboratories, Germany) for 48 h at 37 °C. Virus RNA was extracted, and quality and integrity were controlled using Agilent Technologies 2100 Bioanalyzer (Agilent Technologies; Waldbronn, Germany). A sequencing library was generated from 100 ng total RNA using TotalScript RNA-Seq Kit (epicentre) without fragmentation, according to the manufacturer’s protocol. The libraries were then sequenced on Illumina MiSeq using MiSeq Reagent Kit v2 (500 cycles, paired end runs) and the correct sequence of the re-assortant virus was confirmed. The titer of the stock viruses was determined by focus forming unit (ffu) assay [12].

**Fig. 2:**
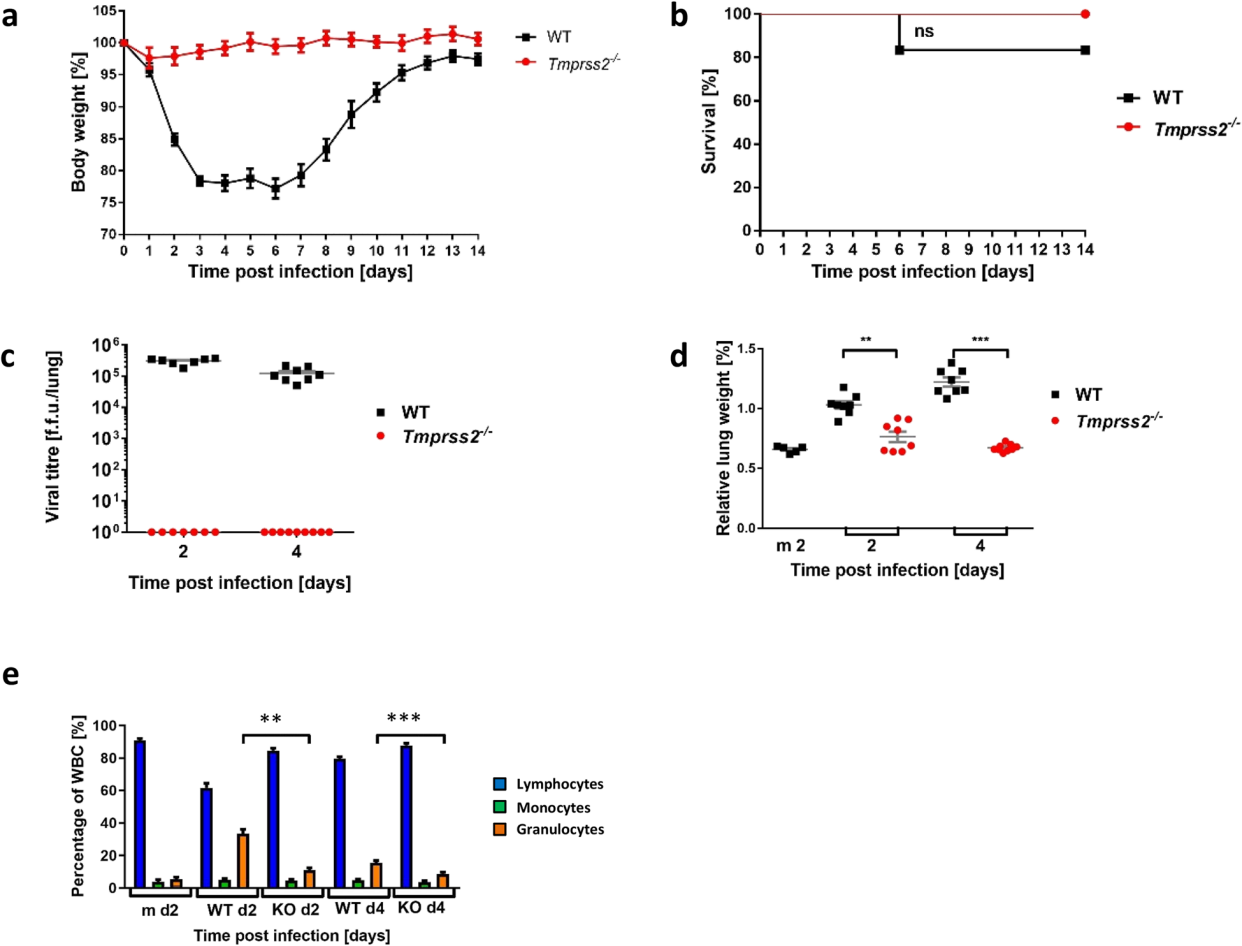
PR8_HA(H2) does not replicate nor cause pathogenesis in *Tmprss2*^*−/−*^ mice. Female C57BL/6 J wild type (WT) and *Tmprss2*^*−/−*^ knock-out (KO) mice (8–12 weeks old) were infected intranasally with 2 × 10^4^ focus forming units (ffu) PR8_HA(H2) (H2N1). Body weight was monitored for 14 days post infection (dpi; WT: *n* = 12; KO: *n* = 10). **a** Mean body weight in percent of starting weight ± 1 standard error of mean (SEM). **b** Survival curve. Statistics for survival curve was calculated with the log rank test. ns: non-significant. **c** Viral load in lung at 2 (WT: *n* = 7; KO: *n* = 7) and 4 dpi (WT: *n* = 8; KO: *n* = 9). Mean titers are shown ±1 SEM. The detection limit for the ffu assay was 40. Undetectable titers were set to 1. **d** Mean (grey) ± 1 SEM of the relative lung weight (lung weight*100/body weight) determined on 2 (WT: *n* = 8; KO: *n* = 8; mock (m2): *n* = 5) and 4 dpi (WT: *n* = 8; KO: *n* = 9). **e** On 2 dpi (WT d2: *n* = 13; KO d2: *n* = 13; mock m d2: *n* = 5) and 4 dpi (WT d4: *n* = 17; KO d4: *n* = 21), eye blood was collected. Cell types (lymphocytes, monocytes and granulocytes) were phenotyped and counted based on cell volume using the VetScan HM5 hematologic system. Cell types are presented in percentage of total white blood cells (WBC). Mock samples were collected from WT mice. To note, we did not compare basal cell counts in KO versus WT since a difference is not expected, although it cannot be excluded. Bars represent mean values, error bars show ±1 SEM. Statistics for (**c**) and (**d**) were calculated by Mann Whitney Tests (** *p* < 0.01; *** *p* < 0.001)

For in vivo studies, female *Tmprss2*^*−/−*^ (B6.129S1-Tmprss2tm1Tsyk) [12, 14] and C57BL/6 JRj wild type (WT) mice (Janvier, 8–12 weeks old) were infected intranasally with 2 × 10^4^ ffu PR8_HA(H2) as described before [12] and changes in body weight and survival were monitored for the next 14 days. Animals with a body weight loss of more than 30% were euthanized and recorded as dead in addition to mice that were found dead. We did not observe dead animals nor significant body weight loss in infected *Tmprss2*^*−/−*^ mice, whereas WT mice exhibited significant body weight loss and some mortality after infection with PR8_HA(H2) virus (Fig. 2a, b). Titers were then determined by ffu assay in each lung as described in [8]. Viral replication in lungs of infected (dose of 2 × 10^4^ ffu) female *Tmprss2*^*−/−*^ and WT mice (8–12 weeks old) revealed no detectable virus replication in *Tmprss2*^*−/−*^ mice, whereas WT mice showed increased lung titers at day 2 and 4 post infection (dpi) (Fig. 2c).

We then investigated other parameters for pathogenicity by measuring relative lung weights, tissue damage and infiltration of immune cells. WT infected mice showed a significant increase in relative lung weights (indicative of hyperemia, pulmonary edema, and/or leukocyte extravasation) compared to *Tmprss2*^*−/−*^ mice that exhibited similar relative lung weights as mock-infected mice (Fig. 2d, e). At 2 and 4 dpi, eye blood was collected and lymphocytes, monocytes and granulocytes were counted using the VetScan HM5 hematologic system as described previously [15]. The analysis of blood cells showed a significant increase of granulocytes in WT mice from 2 to 4 dpi compared to mock infected mice, whereas *Tmprss2*^*−/−*^ mice only showed a marginal increase in granulocytes compared to mock-infected animals (Fig. 2e). An increase of granulocytes is an indicator for the severity of the infection [15].

**Fig. 3:**
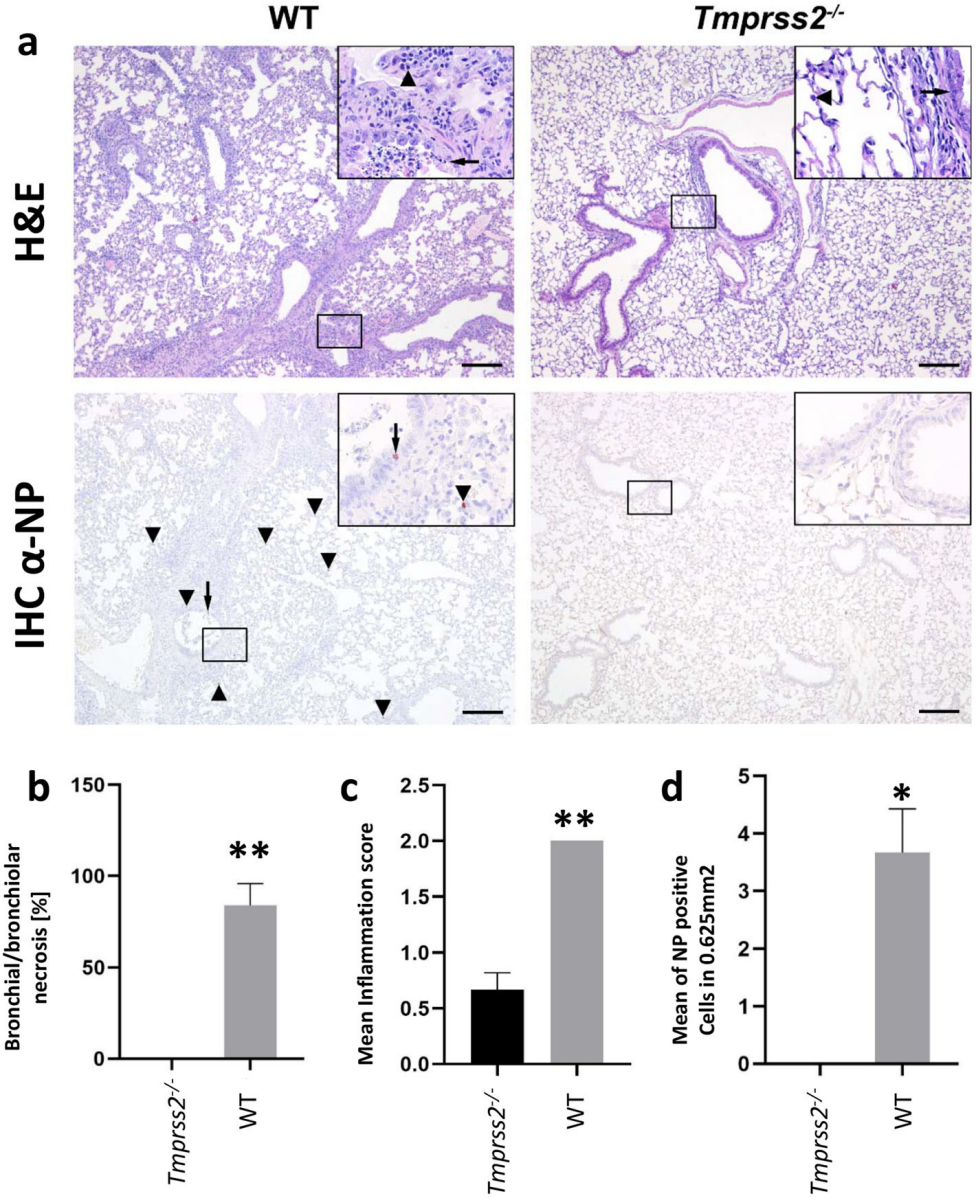
Strongly reduced lung tissue damage, immune cell infiltration and absence of virus replication in infected *Tmprss2*^*−/−*^ mice. Female 8–12-week-old C57BL/6 J wild type (WT) and *Tmprss2*^*−/−*^ knock-out (KO) mice were infected intranasally with 2 × 10^4^ focus forming units (ffu) PR8_HA(H2) (H2N1 virus (WT: *n* = 3; KO: *n* = 3). Lungs were prepared at 4 days post infection (dpi) and paraffin sections were stained with H&E (**a** top). Inserts: arrows point to damaged bronchiolar epithelium; arrowheads indicate mononuclear cells including lymphocytes and macrophages infiltrating lung interstitium and alveolar lumina adjacent to bronchioli. Bars, 200 μm. (**a** bottom) For detection of infected cells by immunohistochemistry, anti-NP (IHC α-NP) antibodies were used and sections were counterstained with Mayer’s hematoxylin. IAV-infected bronchiolar epithelial cells (arrow) and leukocytes (arrowhead) were found in WT mice, whereas no immunostaining was detectable in *Tmprss2*^*−/−*^ mice (see inserts). **b** Necrotic bronchioli were determined as percentage of total cells. **c** Semi quantitative scoring results of cellular infiltration (0 = none, 1 = mild, 2 = moderate, 3 = severe). **d** IAV antigen positive cells were counted twice in 10 randomly selected high power fields (10 × 0.0625 mm^2^). Bars indicate mean values +/− SEM. One-sample t-tests were used for statistical analysis (* *p* < 0.05; ** *p* < 0.01). Please note that all values are identical for WT in the inflammation score (error bar is zero)

We then performed histopathological studies on tissue sections from PR8_HA(H2) infected *Tmprss2*^*−/−*^ and WT mice (Fig. 3). Female 10-week-old WT and *Tmprss2*^*−/−*^ mice were infected intranasally with 2 × 10^4^ ffu PR8_HA(H2). Lungs were prepared at 4 dpi and paraffin sections were stained with hematoxylin and eosin (H&E). Infection was associated with severe bronchiolar epithelial damage and moderate inflammatory cell infiltration (Fig. 3a, b) in WT mice, whereas lungs of *Tmprss2*^*−/−*^ mice exhibited only mild or no damage of bronchiolar epithelium (Fig. 3a, b). Infiltration of mononuclear cells including lymphocytes and macrophages into interstitium and alveolar lumina adjacent to bronchioli was more pronounced in WT compared to *Tmprss2*^*−/−*^ mice (Fig. 3a, c). For immunohistochemical detection of infected cells, a primary antibody against IAV nucleoprotein (EBS-I-238, European Veterinary Laboratory) and a secondary HRP-labeled goat-anti-mouse IgG2a (Southern Biotech) were used [8]. Analysis was performed by counting all IAV-infected cells in 10 randomly selected high power fields (10 × 0.0625 mm^2^) on one immunohistochemically stained section of the complete lung per animal. IAV antigen was detected in bronchiolar epithelial cells and leukocytes of WT mice, whereas in lungs of *Tmprss2*^*−/−*^ mice no antigen positive cells were observed (Fig. 3a, d). This finding corroborates lack of virus detection by ffu titration in lung homogenates at 2 and 4 dpi. Together, these observations showed that *Tmprss2*^*−/−*^ mice were resistant to PR8_HA(H2) virus-induced disease.

We then analyzed cleavage of viral HA from broncho-alveolar lavage (BAL) isolates. Whole lung extracts will contain cleaved and non-cleaved HA that is being produced inside cells. In BAL secretion, mainly viral particles are detected that are released from infected cells. Therefore, BAL is optimal source to test presence of the hemagglutinin and its cleavage state.

**Fig. 4:**
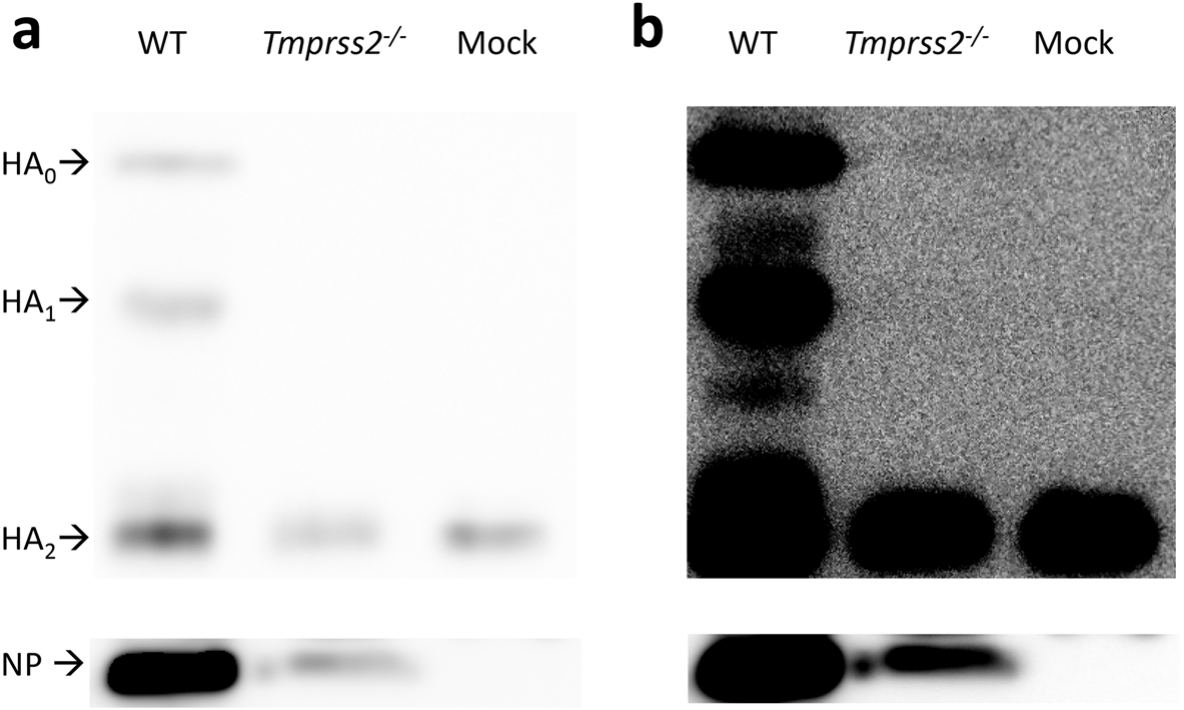
The H2-HA is not processed in *Tmprss2*^*−/−*^ mice. Female 8–12-week-old C57BL/6 J wild type (WT) and *Tmprss2*^*−/−*^ knock-out (KO) mice were infected intranasally with 2 × 10^4^ focus forming units (ffu) PR8_HA(H2). On day 2 post infection (dpi) broncho-alveolar lavages (BAL) were prepared. **a** H2 cleavage and nuclear protein (NP) in BAL (day 2 p.i.) were analysed by Western blots. **b** Enhanced image of (**a**) using ImageJ software (B/C: brightness/contrast). HA_0_: uncleaved HA, HA_1_: N-terminal part of cleaved HA, HA_2_: C-terminal part of cleaved HA; NP: nuclear protein

**Fig. 5:**
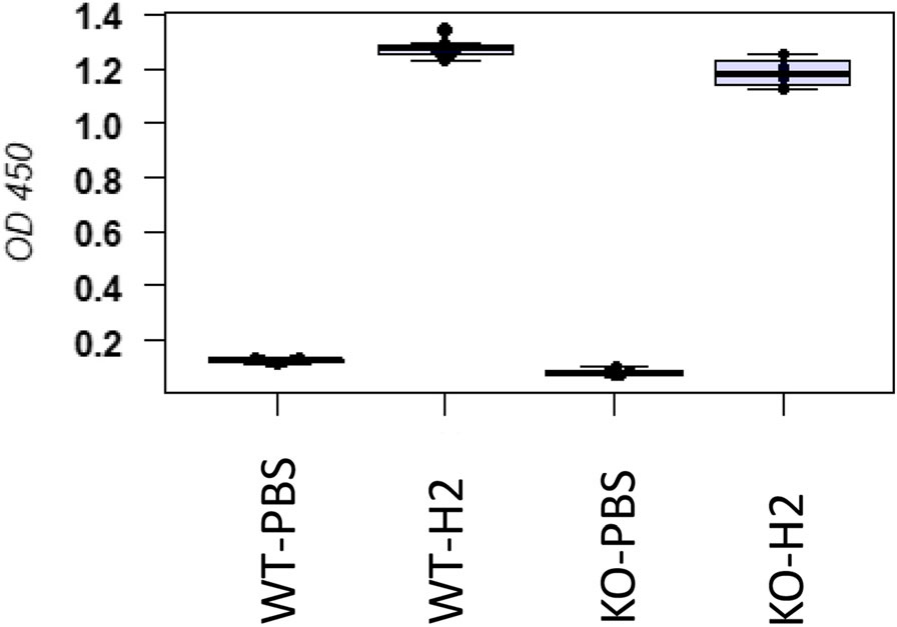
Infected *Tmprss2*^*−/−*^ mice exhibit an adaptive immune response. ELISA of sera from female 8- to 12-week-old PR8_HA(H2) infected wild type (WT) and *Tmprss2*^*−/−*^ knock-out (KO) mice for anti-IAV antibodies. Mice were infected with 2 × 10^4^ focus forming units (ffu) PR8_HA(H2). At 14 days post infection (dpi), sera from infected and PBS mock-treated mice were prepared and analyzed for the presence of IAV-specific antibodies. Boxplots of OD values at 450 nm and median values per group are shown. WT-PBS: PBS treated control WT mice (*n* = 7), WT-H2: WT mice infected with 2 × 10^4^ ffu PR8_HA(H2) virus (*n* = 7), KO-PBS: PBS treated control mice (*n* = 5), KO-H2: KO mice infected with 2 × 10^4^ ffu with PR8_HA(H2) virus (*n* = 4). Differences between the means of treated control and infected mice were significant (*p* < 0.001)

Total protein was determined in BAL samples using the Pierce BCA Protein Assay Kit according to the manufacturer’s instructions and 5 μg protein was run on a SDS-PAGE, blotted on a PVDF-membrane (Bio-Rad), which was then incubated with an anti-H2N2 polyclonal antibody (Sino Biological, 11,688-RP02, HB04DE2907-B) using a dilution of 1:5000 in TBST with 0.1% milk powder and then with an horseradish peroxidase (HRP)-conjugated goat anti-rabbit antibody (Sigma, A0545) in a dilution of 1:100,000. As a reference (internal control) for total viral particles in a sample, we used the viral NP protein. If such a signal is present, viral particles have been produced and viral proteins can be detetcted. Detection of signals was performed using the FujiFilm LAS-3000 imaging system. Subsequently, the membrane was incubated with an anti-NP antibody (GeneTex, GTX125989) in a dilution of 1:10,000, followed by incubation with the same secondary antibody as before. In the BAL of infected WT mice, both HA_1_ and HA_2_ were detectable (Fig. 4). Also, a very faint HA_0_ band was detectable in Tmprss2^−/−^ mice after image enhancement (Fig. 4b). The NP band was much less intense in *Tmprss2*^*−/−*^ mice than in WT mice and only clearly detectable after image enhancement (Fig. 4a, b). Nevertheless, the presence of NP demonstrated that viral particles were present in the samples from *Tmprss2*^*−/−*^ mice that HA_0_ was detected but no cleaved HA_1_. Of note, a band of the size of HA_2_ was observed in BAL of *Tmprss2*^*−/−*^ mice that was also detectable in mock infected lung at approximately the same intensity. Thus, this band in *Tmprss2*^*−/−*^ mice is unspecific and not representing HA_2_. Taken together, these results are consistent with the observed absence of PR8_HA(H2) (H2N1) replication in *Tmprss2*^*−/−*^ mice. Since no viral replication and only minor immune responses were detected in the studies described above, we confirmed that mice had indeed been infected with the PR8_HA(H2) virus by determining the presence of H2-specific antibodies at 14 dpi in the serum. For this, female mice were infected with 2 × 10^4^ ffu PR8_HA(H2). At 14 dpi, sera from infected and PBS mock-treated mice were prepared and analyzed for the presence of IAV-specific antibodies. 96-well plates were coated with 5 × 10^4^ ffu PR8_HA(H2) viruses ml^− 1^, diluted in 1 x PBS (phosphate buffered saline), incubated overnight, washed with PBST (1 x PBS, 0.05% Tween20), and blocked with PBST-FCS (PBST, 5% v/v fetal calf serum). Fifty μl of 1:150 diluted mouse serum was added and incubated for 2 hours (h) at 37 °C. Plates were then washed three times with PBST and 50 μl of anti-mouse IgG (goat anti-mouse IgG heavy chain gamma-HRP, KPL #474–1802), diluted 1:1000 in PBST-FCS, were added and incubated for 2 h at 37 °C. Wells were then washed three times with PBST and 50 μl of substrate (SureBlue TMB Peroxidase Substrate, KPL, #52–00-00) was added. After 3 minutes, the reaction was stopped by adding 50 μl TMB Stop Solution (KPL, #50–85-04) and the optical density was measured at 450 nm. Our studies showed that infected *Tmprss2*^*−/−*^ mice exhibited a significant seroconversion that was similar to infected WT mice (Fig. 5). Thus, incoming virus was able to infect lung cells and trigger an immune response but was not able to spread and replicate to levels detectable in ffu assays. It may be worth noting that in mouse lungs, *Tmprss2* gene expression is down-regulated at the transcriptional level after infection [16].

## Conclusion

We showed that *Tmprss2*^*−/−*^ mice were resistant to H2 IAV pathogenesis. After infection with PR8_HA(H2) *Tmprss2*^*−/−*^ mice did not lose body weight and no viral replication was observed in lungs at 2 and 4 dpi. Histopathological analysis showed strongly reduced inflammatory lesions and the lack of detectable viral antigen in *Tmprss2*^*−/−*^ mice. We and others demonstrated earlier that *Tmprss2*^*−/−*^ mice are also resistant to H1 IAV infection. In these studies, infectious virus was detected at day two after infection in lung homogenates [5, 6, 12]. Thus, some residual replication was observed for H1 virus, whereas in our present study, PR8_HA(H2) virus was not able to replicate to detectable titers in *Tmprss2*^*−/−*^ mice. This difference in detectable virus titers might be due to the origin of the two viruses. The H1-HA viruses (A/Puerto Rico/8/34 or A/California/04/09) were adapted to mouse [5, 6], whereas the HA encoding segment 4 of the H2-HA virus in our study originated from a bird isolate. It is well conceivable that the bird H2-HA is missing adaptations that have been acquired by the mammalian H1-HA and allow for more efficient replication in *Tmprss2*^*−/−*^ mice.

The very low amount of virus particles in *Tmprss2*^*−/−*^ mice precluded the formal demonstration that absence of viral spread and pathogenesis in these animals was due to lack of HA activation. However, several observations support this interpretation: Only a weak NP signal and no HA_1_ signal were detected in samples from Tmprss2^−/−^ animals, even upon overexposure of the immunoblot. Moreover, our studies in cell cultures showed that HA can be cleaved by TMPRSS2 and there is at present no evidence that TMPRSS2 can promote IAV spread by other means than HA cleavage. These points strongly suggest that the absence of proteolytic processing of HA is the underlying cause for the lack of replication and spread of H2-HA IAV in *Tmprss2*^*−/−*^ mice.

In conclusion, our study extends the list of IAV subtypes known to depend on TMPRSS2 for viral spread in mice to the H2 subtype. The present and previous results identify TMPRSS2 as an attractive target for novel anti-IAV drugs.

## Data Availability

All data presented in this manuscript is included in the text.

## Abbreviations

BAL: Broncho-alveolar lavage
dpi: day post infection
FCS: Fetal calf serum
ffu: focus forming unit
HA: Hemagglutinin
H&E: Hematoxylin and eosin
HRP: Horseradish peroxidase
IAV: Influenza A virus
KO: Knock-out
PR8: A/Puerto Rico/8/34 (H1N1)
PBS: Phosphate buffered saline
SPF: Specific pathogen free
TMPRSS2: Transmembrane protease serine 2
WT: Wild type
